# Seroprevalence of Antibodies to *Burkholderia pseudomallei* in Mississippi Gulf Coast Residents, September 2023

**DOI:** 10.3390/pathogens14090921

**Published:** 2025-09-12

**Authors:** Katherine M. DeBord, Mindy G. Elrod, Claire Hartloge, William A. Meyer, Brooke E. Swanson, Caroline A. Schrodt, Maria E. Negron, Zachary P. Weiner

**Affiliations:** 1 Division of High-Consequence Pathogens and Pathology, Centers for Disease Control and Prevention, Atlanta, GA 30329, USA; 2 Quest Diagnostics, Secaucus, NJ 07094, USA

**Keywords:** *Burkholderia pseudomallei*, seroprevalence, communicable diseases, emerging, Mississippi

## Abstract

In 2022, *Burkholderia pseudomallei* was first identified in continental United States (U.S.) environmental samples from the Mississippi Gulf Coast following two autochthonous infections. To better understand the extent of exposure to this emerging bacterium, we tested a convenience sample of 825 residual sera samples (550 from the Mississippi Gulf Coast, 275 from the northern U.S.) from a commercial diagnostic laboratory for the presence of antibodies to *B. pseudomallei*, using an indirect hemagglutination assay. We estimated seroprevalence of antibodies to *B. pseudomallei* in Mississippi Gulf Coast residents and in controls from northern regions of the U.S. where *B. pseudomallei* is less likely to persist in the environment. At a titer cut-off of ≥1:40, we observed a similar seropositivity between Mississippi Gulf Coast residents (14%, 95% CI: 11%, 17%) and controls (17%, 95% CI: 13%, 18%). Similarities in seropositivity suggest environmental exposure to *B. pseudomallei* in the Mississippi Gulf Coast may be limited; however, a lack of accompanying illness and exposure information limits our ability to conclusively interpret these findings. These estimates can serve as a baseline of seropositivity in the U.S. for future studies and to track the spread of *B. pseudomallei* in the U.S. over time.

## 1. Introduction

*Burkholderia pseudomallei* is a Gram-negative bacterium and Tier 1 select agent that causes melioidosis, a rare but serious disease [1]. *B. pseudomallei* typically lives in soil and water in regions with tropical and subtropical climates around the world, such as South and Southeast Asia, northern Australia, parts of Central and South America, the United States (U.S.) Virgin Islands, and Puerto Rico. It can infect both animals and humans through direct contact with skin (especially through cuts or wounds) or mucous membranes, inhalation, or ingestion. In regions where melioidosis is highly endemic, such as Thailand and Northern Australia, most healthy people who are exposed to *B. pseudomallei* never develop melioidosis [2,3]. One study from Thailand estimated only 1 in 4600 antibody-producing exposures resulted in clinical infection [3]. In people who do develop disease, symptoms can be highly variable and non-specific, often leading to mis- or under-diagnosis [4,5]. Over half of melioidosis cases present as pneumonia; other common presentations include sepsis, abscess formation (prostate, spleen, liver, and kidney), and genitourinary, skin and soft tissue, musculoskeletal, and neurological involvement [4]. Mortality can range from 14 to 43% [4].

In 2022, the Centers for Disease Control and Prevention (CDC) identified *B. pseudomallei* for the first time in the environment in the continental U.S. [6]. *B. pseudomallei* was identified through environmental sampling of soil and water in the Gulf Coast region of southern Mississippi during an investigation of two human melioidosis cases with no travel history. It is unclear how long the bacterium has been in the environment prior to the first case in 2020 or how widespread the bacterium is in the continental U.S. Modeling suggests that the environmental conditions found in the Gulf Coast states, such as warm, moist soil with a pH of 4–8, are conducive to the growth of *B. pseudomallei* [7,8].

Detection of antibodies to *B. pseudomallei* in blood samples using a serologic assay can reflect prior exposure [9]. Because clinical disease following exposure is rare, and illnesses may be mis-diagnosed when they do occur, seroprevalence studies in newly endemic areas may help to better define the geographic distribution of *B. pseudomallei* in a region. To better understand the extent of exposure to this emerging bacterium in the Gulf Coast of the U.S., we used convenience samples of residual clinical specimens from a commercial diagnostic laboratory to conduct a serologic survey in the Gulf Coast region of Mississippi. We estimated seroprevalence of antibodies to *B. pseudomallei* by age groups in the Gulf Coast region of Mississippi and compared it to seroprevalence seen in northern regions of the U.S., which are less likely to support persistence of *B. pseudomallei* in the environment.

## 2. Materials and Methods

### 2.1. Sample Selection

We obtained a convenience sample of deidentified residual patient sera collected for routine screening (e.g., cholesterol screening) or clinical management by a commercial diagnostic laboratory. Samples were selected for inclusion based on reported patient zip code of residence and available residual sera volume (≥0.3 mL). Samples meeting inclusion criteria were pulled and stored prospectively in September 2023 until the study sample size was met (550 Mississippi residents and 275 controls). Mississippi Gulf Coast residents were defined as patients with reported residence in the 47 zip codes that make up six Mississippi counties: Hancock, Harrison, Jackson, Pearl River, Stone, and George. Controls were patients who visited a laboratory in the Chicago, Illinois, area but did not have a reported residence zip code within Chicago city limits. The northern U.S., including Illinois, is considered to be an area of low suitability for *B. pseudomallei* persistence in the environment [8]. Residents from the City of Chicago were excluded to better align with population demographics of the six Mississippi counties selected for this study.

### 2.2. Laboratory Testing

Serology was performed at CDC using the *B. pseudomallei* indirect hemagglutination assay (IHA) following standard CDC protocols [10]. Pooled antigens were separately prepared from two clinical *B. pseudomallei* isolates: strain PM42 from northeast Thailand (Thailand Ministry of Public Health, Bangkok, Thailand) and strain MSHR 465a from Northern Australia (Menzies School of Health Research, Darwin, Australia). These strains were selected from two highly endemic regions and represent commonly circulating strains. The antigens were prepared in-house using killed whole cell lysates; the process is detailed in the standard operating procedure developed by the Mahidol-Oxford Research Unit (MORU) [11]. Performance of the in-house IHA has been validated by CDC using true positives (specimens from culture confirmed cases) and true negatives (melioidosis-rule out, specimens confirmed as another pathogen, and healthy volunteers). We defined seropositivity as a single titer value of ≥1:40.

### 2.3. Statistical Analysis

We calculated seroprevalence as the proportion of specimens that were classified as seropositive stratified by geographic area and assumed the variance had a negative binomial distribution to calculate 95% confidence intervals (95% CIs). We compared estimated seroprevalence from residents in the Mississippi Gulf Coast to estimated seroprevalence among control residents using chi-square tests and two-sided *p* values less than 0.05 to define statistical significance. We also calculated age-standardized seroprevalence estimates using weights derived from U.S. Census Bureau 2023 county-level population estimates for the 6 counties in Mississippi Gulf Coast [12]. We conducted sensitivity analyses using more conservative titer cut-off values (≥1:160 and ≥1:320) to define seropositivity. Statistical analyses were conducted in SAS version 9.4 (Cary, NC, USA) and maps were generated in QGIS version 3.34.1.

### 2.4. Ethics Statement

Ethical review and approval were waived for this study as all specimens were de-identified without the ability to link test results back to specific individuals.

## 3. Results

**Figure 1:**
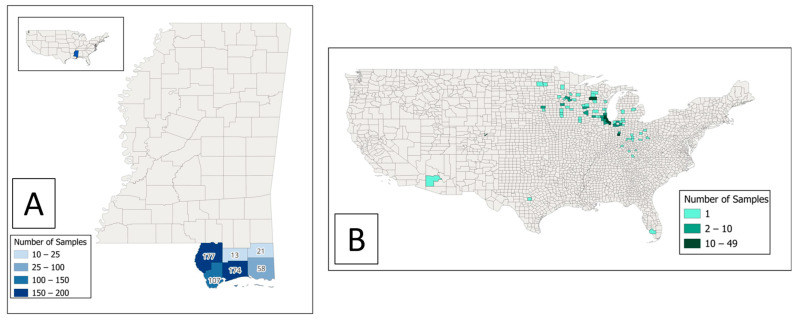
Geographic distribution of residence county for residual sera samples tested for *Burkholderia pseudomallei* seropositivity among (**A**) Mississippi Gulf Coast residents and (**B**) controls.

We tested 825 residual sera samples, 550 from Mississippi Gulf Coast residents and 275 from the Chicago area. The geographic distribution of residence by county for the 825 individuals is displayed in Figure 1. All specimens were collected from 12 to 29 September 2023. Of all the specimens, 52% (432/825) were from female patients and the median patient age was 59 [interquartile range (IQR): 45, 69] years (Table 1). The sex and age distribution were similar between Mississippi Gulf Coast residents and controls.

At a titer cut-off of ≥1:40, we observed a similar seropositivity between Mississippi Gulf Coast residents (14%, 95% CI: 11%, 17%) and controls (17%, 95% CI: 13%, 18%) (*p* = 0.3; Table 1). Among those who were seropositive, IHA titers ranged from 1:40 to 1:5120. Four individuals had titers ≥1:640. The trend of similar seropositivity between Mississippi Gulf Coast residents and controls persisted even with more conservative definitions of seropositivity.

**Figure 2:**
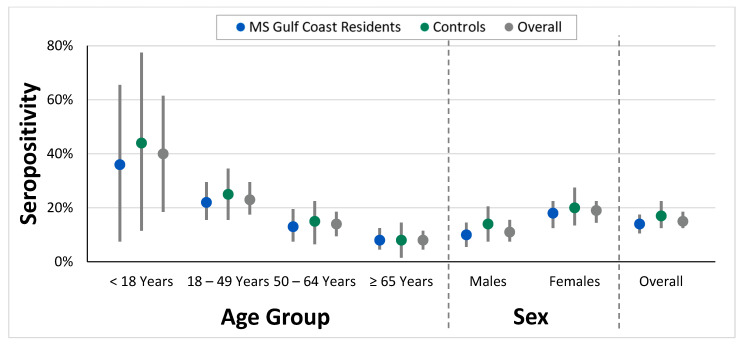
Seropositivity to *Burkholderia pseudomallei* antibodies and 95% confidence intervals (denoted by grey bars) by age group, sex, and geographic location.

Observed seropositivity decreased from younger to older age groups for both the Mississippi Gulf Coast residents and controls, ranging from 40% (95% CI: 19%, 61%) seropositive in individuals <18 years of age to 8% (95% CI: 5%, 11%) in individuals ≥65 years of age (Figure 2). Very few specimens from individuals aged <18 years (20/825, 2%) led to large confidence intervals for these age groups. When age-standardized, the seropositivity for Mississippi Gulf Coast residents (21%, 95% CI: 18%, 25%) was observed to be slightly higher than the crude seropositivity in this population (14%, 95% CI: 11%, 17%). Slightly higher rates of seropositivity were observed among females (19%, 95% CI: 15%, 22%) compared to males (11%, 95% CI: 8%, 15%) overall. Similar trends were seen in both the Mississippi Gulf Coast residents and controls; however, the confidence intervals overlapped, suggesting the difference may not be a true difference.

## 4. Discussion

We observed similar seroprevalence of antibodies to *B. pseudomallei* between Mississippi Gulf Coast residents and controls from the northern regions of the U.S., suggesting no differences in environmental exposures between these two groups. These estimates represent the first published estimates of *B. pseudomallei* seropositivity in U.S. populations.

While our estimates of *B. pseudomallei* seropositivity in Mississippi Gulf Coast residents and the control group were similar, they were also non-zero, despite the northern U.S. being considered unsuitable for *B. pseudomallei* growth in the environment [8]. This may be due to individuals being exposed to *B. pseudomallei* in the environment while traveling to or living in a country where *B. pseudomallei* is known to be endemic. Travel history of individuals included in this study is not known; however, 14% of the U.S. population are foreign-born [13] and 76% of the U.S. population have traveled internationally [14], which may contribute to increasing the background seropositivity. False-positive reactions resulting from cross-reactivity of the IHA to other less virulent *Burkholderia* species like *B. thailandensis* may be a factor as well [15]. *B. thailandensis* has been identified in environmental samples from the southern U.S.; however, its full geographic distribution in the Western Hemisphere is unknown [16].

Published estimates of *B. pseudomallei* seroprevalence in regions where *B. pseudomallei* is known to be endemic vary widely (3.1%–81%) [2,9,17,18,19]. This variability may be due to differences in assay development [20], definitions of seropositivity [21], prevalence of the bacterium in the environment, climate, and mechanisms by which different populations are exposed to the bacterium. Our overall estimates of seroprevalence (15%, 95% CI: 13%, 18%) are higher than seroprevalence estimates from endemic parts of northern Australia (3.1%) [2], but are much lower than estimates from Thailand (38%) [9] and northeastern Brazil (81%) [18]. Having estimates of *B. pseudomallei* seroprevalence in a U.S. population using a CDC-standardized protocol can serve as a baseline of seropositivity in the U.S. for future studies.

We observed an inverse relationship between age and seropositivity among both Mississippi Gulf Coast residents and controls, with the highest seropositivity rates among persons <18 years of age and the lowest seropositivity rates among persons ≥65 years of age. This may be due to the natural waning of overall antibody levels as a person ages or potential increased rates of immuno-compromising conditions in the older age groups in the study, which could suppress immune response. Future seroprevalence studies could explore this inverse relationship further by capturing risk factor information, such as information on immune-compromising conditions and medications, as well as including more individuals from younger age groups.

We identified four individuals (1 Mississippi Gulf Coast resident and 3 controls) with titers ≥1:640, which is generally regarded as indicative of a current or prior *B. pseudomallei* infection. In a *B. pseudomallei* endemic region of Australia, 61% of individuals with IHA titers ≥1:640 had culture confirmed melioidosis [22]. Our dataset had no accompanying clinical information, so we are unable to say if these four individuals were actively being managed for infection or if they had an undiagnosed infection in the past. Given the non-specific symptoms, providers should consider melioidosis as a differential if the patient has a compatible illness and travel history to a *B. pseudomallei* endemic area including the Mississippi Gulf Coast.

There are several limitations to this study to acknowledge. The specimens used were a convenience sample of specimens collected for clinical purposes from persons seeking healthcare either for an acute illness or as part of routine care. Residual clinical specimens from screening or routine care are more likely to come from persons who are older or require monitoring for chronic conditions. This was seen in our data where the median age of Mississippi Gulf Coast residents included in this study was 59 years compared to a median age of 39 years for the general Mississippi population [12]. Further, specimens were shared with CDC with limited accompanying data. No data on recent symptomatic illness, potential high-risk exposures, or travel to melioidosis-endemic countries were available, hindering our ability to fully assess the individual’s risk of infection. IHA results can be challenging to interpret as the assay may cross react with other *Burkholderia* spp. leading to false positive results. Also, some patients with confirmed melioidosis never develop detectable titers leading to lower rates of seropositivity [4]. We tried to mitigate these limitations by using a control group. Future studies could compare results from IHA and a validated Enzyme-Linked Immunosorbent Assay (ELISA) to better interpret sensitivity and specificity of IHA in this population. Additionally, these specimens were single-point-in-time collections with no ability to follow-up with individuals, especially those with higher titers, to determine if the individuals developed melioidosis. Lastly, we were only able to test 825 samples collected during a two-week time frame due to limited resources. Future studies could include larger sample sizes collected at different time points throughout the year to decrease confidence intervals and look for seasonal patterns in seropositivity.

## 5. Conclusions

Similarities in seropositivity between Mississippi Gulf Coast residents and controls from northern regions of the U.S. suggest that environmental exposure to *B. pseudomallei* in the Mississippi Gulf Coast may be limited; however, the lack of accompanying illness and exposure information limits our ability to conclusively interpret these findings. Despite this limitation, these estimates can serve as a baseline of seropositivity in the U.S. for future studies and to track the spread of *B. pseudomallei* over time in the U.S.

## Figures and Tables

**Table 1:** Demographic information and serology results for individuals with residual sera samples tested for *Burkholderia pseudomallei* seropositivity.

	MS ^1^ Gulf Coast Residents N = 550	Controls N = 275	Overall N = 825	*p*-Value
Demographic Information				
Sex, n (%)				
Male	225 (46)	138 (50)	393 (48)	0.3
Female	295 (54)	137 (50)	432 (52)	
Age in years, median (IQR ^2^)	59 (47, 70)	58 (41, 67)	59 (45, 69)	0.01
Age groups, n (%)				
<18 years	11 (2)	9 (3)	20 (2)	0.06
18–49 years	154 (28)	91 (33)	245 (30)	
50–64 years	163 (30)	89 (32)	252 (31)	
≥65 years	222 (40)	86 (31)	308 (37)	
Serology Results				
Titers, n (%)				
<1:10	360 (65)	154 (56)	514 (62)	0.07
1:10	58 (11)	44 (16)	102 (12)	
1:20	54 (10)	30 (11)	84 (10)	
1:40	45 (8)	19 (7)	64 (8)	
1:80	17 (3)	16 (6)	33 (4)	
1:160	10 (2)	7 (3)	17 (2)	
1:320	5 (1)	2 (1)	7 (1)	
1:640	1 (0.2)	1 (0.4)	2 (0.2)	
1:1280	0 (0)	1 (0.4)	1 (0.1)	
1:5120	0 (0)	1 (0.4)	1 (0.1)	
Seropositivity, n (%, 95% CI ^3^)				
≥1:40	78 (14, 11–17)	47 (17, 13–22)	125 (15, 13–18)	0.3
≥1:160	16 (3, 2–4)	12 (4, 2–7)	28 (3, 2–5)	0.3
≥1:320	6 (1, 0.2–2)	5 (2, 0.2–3)	11 (1, 0.6–2)	0.5

^1^ MS: Mississippi, ^2^ IQR: interquartile range, ^3^ CI: confidence intervals.

## Data Availability

The raw data supporting the conclusions of this article will be made available by the authors on request.

## Abbreviations

The following abbreviations are used in this manuscript:

CDC	Centers for Disease Control and Prevention
CI	Confidence intervals
IHA	Indirect hemagglutination assay
IQR	Interquartile range
MS	Mississippi
U.S.	United States
